# Mental disorders and their impact on academic performance in Australia: a matched population-based cohort study.

**DOI:** 10.23889/ijpds.v7i3.1779

**Published:** 2022-08-25

**Authors:** Rebecca Mitchell, Anne McMaugh, Carolyn Schniering, Cate Cameron, Reidar Lystad, Tim Badgery-Parker, Olav Nielssen

**Affiliations:** 1 Australian Institute of Health Innovation, Macquarie University; 2 Macquarie University; 3 Jamieson Trauma Institute

## Objectives

To compare scholastic performance and high school completion of young people hospitalised with a mental disorder compared to young people not hospitalised for a mental disorder by sex.

## Approach

A population-based matched case-comparison cohort study of young people aged ≤18 years hospitalised for a mental disorder during 2005-2018 in New South Wales, Australia using linked birth, health, education and mortality records. The comparison cohort was matched on age, sex and residential postcode. Generalised linear mixed modelling examined risk of school performance below the national minimum standard (NMS) and generalised linear regression examined risk of not completing high school for young people with a mental disorder compared to matched peers.

## Results

Young males with a mental disorder had over a 1.7 times higher risk of not achieving the NMS for numeracy (ARR: 1.71; 95%CI 1.35-2.15) and reading (ARR: 1.99; 95%CI 1.80-2.20) compared to matched peers. Young females with a mental disorder had around 1.5 times higher risk of not achieving the NMS for numeracy (ARR: 1.50; 95%CI 1.14-1.96) compared to matched peers. Both young males and females with a disorder had around a three times higher risk of not completing high school compared to peers. Young males with multiple disorders had up to a six-fold increased risk and young females with multiple disorders had up to an eight-fold increased risk of not completing high school compared to peers.

## Conclusion

Early recognition and support could improve school performance and educational outcomes for young people who were hospitalised with a mental disorder. This support should be provided in conjunction with access to mental health services and school involvement and assistance.

